# Novel Polymorph of Favipiravir—An Antiviral Medication

**DOI:** 10.3390/pharmaceutics13020139

**Published:** 2021-01-21

**Authors:** Alexander S. Goloveshkin, Alexander A. Korlyukov, Anna V. Vologzhanina

**Affiliations:** 1 A. N. Nesmeyanov Institute of Organoelement Compounds of the Russian Academy of Sciences, 28 Vavilova str, 119991 Moscow, Russia; golov-1@mail.ru (A.S.G.); alex@xrlab.ineos.ac.ru (A.A.K.); 2 Higher Chemical College of the Russian Academy of Sciences, D.M. Mendeleev University of Chemical Technology of Russia, Miusskaya sq. 9, 125047 Moscow, Russia

**Keywords:** active pharmaceutical ingredient, quantum theory “atoms in molecules”, polymorphism, powder X-ray diffraction, periodic density functional theory calculations

## Abstract

Various solid forms of pharmaceutically important compounds exhibit different physical properties and bioactivity; thus, knowledge of the structural landscape and prediction of spontaneous polymorph transformations for an active pharmaceutical ingredient is of practical value for the pharmaceutical industry. By recrystallization from ethyl acetate, a novel polymorph of 6-fluoro-3-hydroxypyrazine-2-carboxamide (trademark favipiravir, RNA polymerase inhibitor) was obtained and characterized using differential scanning calorimetry (DSC), infra-red spectroscopy and powder X-ray diffraction (XRD) analysis. The favipiravir molecule in two polymorphs realizes similar H-bonding motifs, but the overall H-bonded networks differ. Based on periodic density functional theory calculations, the novel tetragonal polymorph with two interpenetrated H-bonded networks is slightly less stable than the orthorhombic one with the **zst** topology of the underlying H-bonded net that is in accord with experimentally observed powder XRD patterns of slow conversion of the tetragonal phase to the orthorhombic one. However, topological analysis of net relations revealed that no transformations can be applied to convert H-bonded networks in the experimental unit cells, and DSC data indicate no solid-state reactions at heating.

## 1. Introduction

A detailed understanding of polymorphism is of crucial importance for many applications in a range of industries due to the prominent variation in properties of solids of the same chemical formula but with different crystal packing. Particularly in the pharmaceutical industry, different polymorphs of an active pharmaceutical ingredient (API) are known to exhibit different solubility, hygroscopicity, tabletability, bioavailability, et cetera [1,2,3,4,5,6,7,8]. Polymorphism at ambient conditions can be associated with various conformations of a flexible API, various molecular packing or with different systems of hydrogen bonds for polytopic molecules. In the latter case, either the local molecular environment can differ as a result of competition between various functional groups, or topological isomers of H-bonded networks can appear for molecules with similar local environments.

**Scheme 1:**
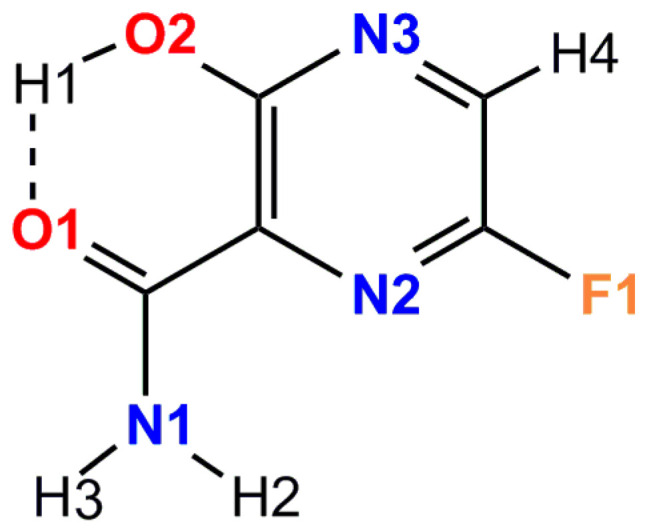
Favipiravir molecule with atom numbering. Intramolecular OH...O bond is depicted with dashed line.

During our study devoted to the screening of novel solid forms of active pharmaceutical ingredient, a novel polymorph of favipiravir (Scheme 1) was obtained. This compound is used as antiviral medication against Ebola virus [9], Lassa virus [10] and COVID-19 [11,12]. Herein, we report on the crystal structure of the novel polymorph of favipiravir as obtained from powder X-ray diffraction data, its infra-red spectra and calorimetric data. A comparison of the local molecular environment and overall packing was carried out. The absence of direct transformations between two H-bonded networks was demonstrated.

## 2. Materials and Methods

Favipiravir substance was obtained from Nantong Jinghua Pharmaceutical Co., Ltd. (Nantong, China) and recrystallized from ethyl acetate. IR spectra of solids were recorded on an IR spectrometer with a Fourier transformer Shimadzu IRTracer100 (Kyoto, Japan) in the range of 4000–600 cm^−1^ at a resolution of 1 cm^−1^ (Nujol mull, KBr pellets). The calorimetric studies were performed on a Discovery DSC 25 calorimeter (TA Instruments, New Castle, DE, USA). The heating and cooling rates were both equal to 5 °C/min.

### 2.1. Powder XRD Studies

The powder patterns were measured using a Bruker D8 Advance (λ(CuKα) = 1.5418 Å, Ni filter, Bragg–Brentano geometry, 1D-detector LynxEye, Bruker AXS, Inc., Madison, WI, USA) diffractometer, θ/θ scan from 5.0° to 50°, step size 0.020°, with the sample deposited on a silicon zero background holder. The pattern of the new form was indexed using the SVD-Index [13] as implemented in TOPAS 5 software [14]. The model for the solution and refinement was prepared based on a structure of the orthorhombic modification. The solution was obtained using the Parallel Tempering method as implemented in FOX [15]. The position of the favipiravir molecule in the new tetragonal modification was then refined using rigid body and Rietveld refinement in TOPAS 5 [14]. The atomic coordinates of the orthorhombic modification in the final refinement were not refined.

Crystal Data for C_5_H_4_FN_3_O_2_ (M = 157.11 g/mol): space group P4_2_/n (no. 86), a = 9.2805(7) Å, c = 14.6491(13) Å, V = 1261.7(2) Å^3^, Z = 8, T = 298 K, D_calc_ = 1.657 g/cm^3^ (Table 1). The Rietveld refinement converged to R_p_/R_P’_/R_WP_/R_WP’_/R_Bragg_ values of 3.79/10.23/5.19/10.13/1.48% with R_exp_/R_exp’_ of 2.35/4.59%, GOF = 2.21. The crystallographic data for the new tetragonal form were deposited in CCDC № 2047143.

### 2.2. DFT Calculations

All DFT calculations of the crystal structures of polymorphs were performed within the PBE exchange–correlation functional using VASP 5.4.1 [16,17,18,19]. Atomic coordinates were optimized; however, cell parameters were fixed at their experimental values to prevent cell contraction or expansion. To improve the description of van-der-Waals interactions, D3 correction [20] was applied. Atomic cores were described using small-core PAW potentials. Valence electrons (2s and 2p for O and N atoms; 3p, and 3s for S; 1s for H) were described in terms of a plane-wave basis set (the kinetic energy cutoff was 1000 eV). Atomic coordinates of optimized structures are given as Supplementary material S1. The vibrational contribution was not considered because its evaluation is extremely time-consuming. Charge density studies were carried out using the AIM program (a part of ABINIT software [21]). The energies of intermolecular interactions were estimated with the formula proposed by Espinosa, Mollins and Lecomte (EML) for hydrogen bonds [22].

## 3. Results

### 3.1. Crystallization and Characterization

6-Fluoro-3-hydroxypyrazine-2-carboxamide commercially named favipiravir is an inhibitor of viral RNA polymerase; it is active against RNA viruses in vitro and in vivo [23]. A four-step synthesis and crystal structure of favipiravir were published by F. Shi et al. in 2014 [24]. In orthorhombic form, the molecule is nearly planar due to intramolecular O-H...O bonding, and is involved in intermolecular H-bonding and aromatic stacking.

**Figure 1:**
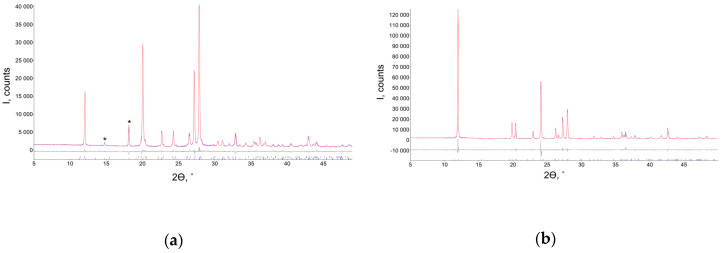
Powder XRD patterns for new tetragonal (**a**) and orthorhombic (**b**) forms of favipiravir. Experimental (blue), calculated (red) and their difference (grey) curves are depicted. The asterisks denote characteristic peaks of the new tetragonal form (111 and 112 reflections).

**Figure 2:**
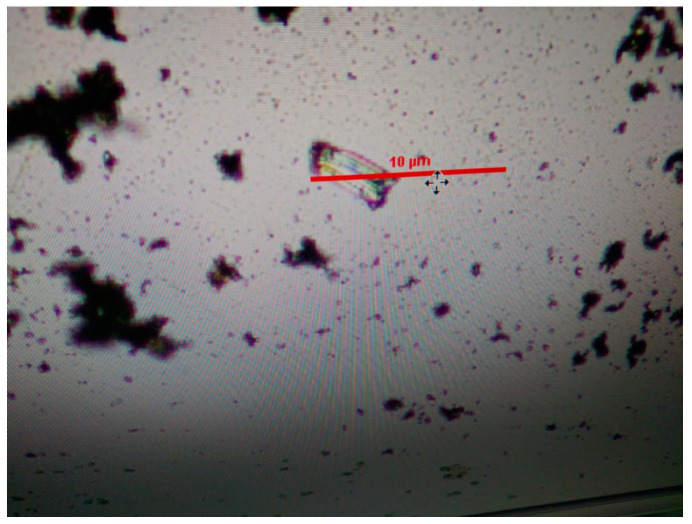
Microphotography of favipiravir recrystallized from ethyl acetate. A single crystal in the center corresponds to the impurity of the orthorhombic phase in mass of the tetragonal one.

By recrystallization of the orthorhombic form from ethyl acetate, a novel polymorph was obtained from powder X-ray diffraction data (Figure 1). The pattern was indexed in the tetragonal P4_2_/n space group, and the crystal structure of the novel polymorph was solved and refined using powder XRD data. The unit cell parameters of the tetragonal and orthorhombic modifications demonstrated some similarities: *a_tetra_* ≈ *a_orth_* ≈ *2c_orth_, c_tetra_* ≈ *b_orth_*. That is why the peaks of two modifications partly overlap. However, the new modification has two characteristic peaks at 2θ = 14.8 and 18.1°. Their presence allows one to reveal the formation of this form even without refining the powder pattern. Note that the pure orthorhombic form was obtained from methanol, ethanol, iso-propanole, tert-butanole, acetone, acetic acid, acetonitrile, butylacetate, toluene and water. No crystals of the tetragonal polymorph suitable for single crystal X-ray diffraction could be obtained (Figure 2), as their sizes were less than 1μm. These results are in accord with the crystallite size for the tetragonal phase estimated from powder XRD data using the Rietveld refinement as 107(5) nm.

The tetragonal polymorph was additionally characterized by means of infra-red (IR) spectra and differential scanning calorimetry (DSC) depicted in Supplementary Figures S1 and S2, respectively. In both IR spectra, the most intense band is situated in the 1380-1450 cm^−1^ region and corresponds to the ν(C-F) vibrations. Intensities of the sharp bands at c.a. 1275 and 1650 cm^−1^ corresponding to the ν(C-OH) and ν(C=O) vibrations are higher for the tetragonal polymorph, while intensities of bands in the range 1550–1630 cm^−1^ indicative of the N-C=O group remain nearly unchanged.

DSC curves for both polymorphs were measured from −50 to 210°C (Figure S2, ESI). Tetragonal and orthorhombic polymorphs melt at, respectively, 192.5 and 190.7°C with partial decomposition. No peaks corresponding to phase transitions at heating or cooling were observed; thus, temperature-induced phase1-to-phase2 transformations between these polymorphs are absent. For both substances, amorphous solids were obtained from melt.

### 3.2. Crystal Structure of the Tetragonal Polymorph

**Figure 3:**
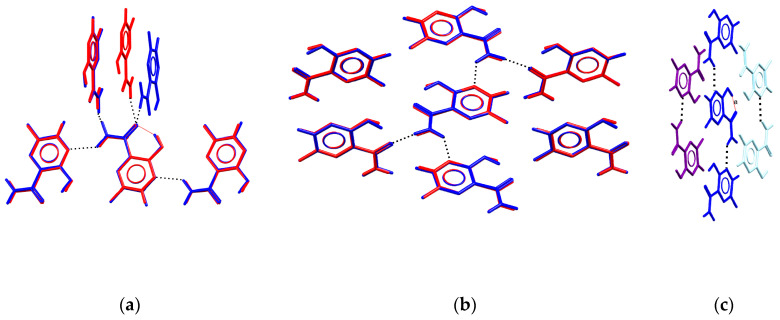
(**a**,**b**) Comparison of local environment of favipiravir in orthorhombic (red) and tetragonal (blue) polymorphs. (**c**) Side view of parallel motifs in the tetragonal polymorph of favipiravir. H-bonds are shown with dashed lines.

The asymmetric unit of the tetragonal polymorph contains one molecule of the title compound. The planar molecular conformation is supported with the intramolecular O-H...O bonding between hydroxide and amide groups. Additionally, each molecule takes part in intermolecular H-bonding with four neighboring molecules. Hydrogen atoms of the amide group interact with oxygen atoms of the amide group, and N(4) atoms of the pyrazine cycle. In both polymorphs, the local environment is similar. For three of the four neighbors, the average deviation of non-hydrogen atoms is less than 0.1 Å, and only the disposition of the acceptor of H-bonding towards the oxygen atom of the amide group differs for the two polymorphs (Figure 3a). Comparison of two crystal structures using the ”Crystal Packing Similarity” tool [25,26] indicates clusters of seven molecules with an average deviation of non-hydrogen atoms of 0.08 Å only (Figure 3b) connected through H-bonds, C-H...O and C-H...F interactions. The H-bonded chains formed by N-H...N interactions are packed parallel to the distance between their meanplanes equal to 3.17–3.31 Å (Figure 3c). Thus, molecular conformations of favipiravir in the two polymorphs are similar, and in both solids, the molecule can be considered as a four-connected node of a network formed by hydrogen bonds. Note that in accord with the Etter rules [27,28], the molecule should tend to form all the possible hydrogen bonds. As the molecule realizes the intramolecular O-H...O bond, we expect that a pure favipiravir will be involved in four strong hydrogen bonds (through two donor hydrogen atoms of the amide group, and any two of the four remaining acceptor atoms, N2, N3, O1 or O2). However, possible competition between various functional groups arouses the question as to whether the experimentally observed local H-bonded environment corresponds to the most likely H-bonds, and if the OH group can also form intermolecular interactions.

**Figure 4:**
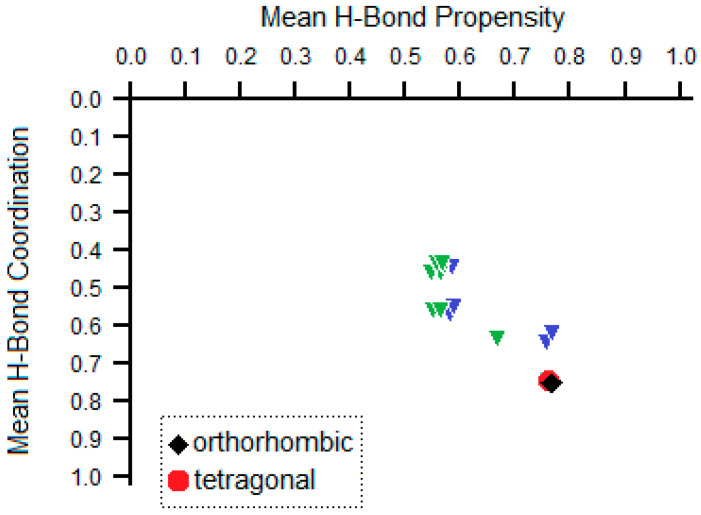
The putative structure landscape for favipiravir, expressed as mean H-bond coordination and propensity for different sets of H-bonds. Theoretically calculated sets of H-bonds are shown with triangles (blue and green correspond to 2 and 3 H-bond pairs for a molecule, respectively). The sets of H-bonds for actual orthorhombic (refcode {DOHVED} in the Cambridge Structural Database [30]) and tetragonal (optimized at VESTA) polymorphs are depicted in the black diamond and red circle, respectively.

Recently, a convenient tool for analysis of the competition of H-bonding donors and acceptors in crystals of polyfunctional molecules was suggested [29]. The tool is based on occurrences of various H-bonded synthons in the Cambridge Structural Database containing more than 1,000,000 crystal structures of small organic and organometallic compounds [30]. The tool was previously applied for analysis of H-bonding systems in some drug molecules [31,32,33,34,35,36,37,38]. The results of ranking synthons by propensity for the favipiravir molecule are listed in Table 2. The favipiravir molecule contains four functional groups (amide, two aromatic nitrogens and hydroxide) that contain, in total, three donor hydrogen atoms (from amide and hydroxide) and four acceptors (oxygen atoms of amide and hydroxide, two aromatic nitrogen atoms) of H-bonding. Thus, the solvent-free form of favipiravir can theoretically realize eleven different types of hydrogen bonds. These bonds are ranked using the H-bonding propensity tool implemented within the Mercury package [39] as listed in Table 1. Among them, the amide…pyrazine NH…N and amide…amide NH…O hydrogen bond are the most likely intermolecular interactions to occur. The hydroxide group is more likely to take part in intramolecular H-bonding with the oxygen atom of the amide group. Experimentally observed H-bonds in both polymorphs are in accord with the results of this calculation, indicating that other theoretically possible polymorphs with another H-bonding environment would be less stable than experimentally observed ones. This conclusion is visualized in Figure 4, where a possible H-bonded polymorphism is depicted as a set of green and blue triangles. Green and blue denote the overall number of H-bond pairs of a molecule (3 and 2, respectively), so that the higher the overall number of H-bonds and of H-bond pairs, the lower a point is situated on the putative structure landscape. For each set of H-bond pairs, their mean H-bond propensities were also calculated based on values in Table 2. Thus, the higher the mean propensity is, the more a point is shifted to the right. Thus, the points that correspond to a molecule that realizes the maximal number of H-bonds with the highest propensities are situated in the bottom right corner of Figure 4 and are expected to correspond to the most stable polymorph. Despite variations in the overall packing, positions of H-bonding environments for both polymorphs of favipiravir correspond to the most stable bonding. Thus, additional calculations of crystal packing energy are required to reveal which of the two polymorphs is more stable. 

### 3.3. DFT Calculations and Charge Density Studies

Detailed inspection of orthorhombic and tetragonal polymorphs revealed that structural data were obtained in noticeably different experimental conditions (single crystal and powder XRD, respectively, different resolution, etc.); however, temperature was almost the same. The optimization in experimental parameters using high cutoff for plane wave expansion (1000 eV) and hard PAWs with small cores described by pseudowave function indicated that the orthorhombic polymorph is 1.80 kJ/mol more stable that the tetragonal one. To compare the structures of the polymorphs, we decided to optimize both cell parameters and atomic coordinates. As a result, the entirely relaxed structures of polymorphs at 0 K were obtained. Unfortunately, the final structure of the polymorphs was affected by uncertainties of DFT theory, i.e., wrong treatment of dispersion interactions that are crucial, particularly for cell parameters. To partly compensate for such uncertainties, the empirical dispersion corrections were utilized; the popular and robust choice was Grimme’s D3 correction [20]. Despite the D3 correction used for dispersion interactions, cell expansion was observed in optimized structures of both polymorphs. The density of tetragonal polymorphs appeared to be slightly less than that for orthorhombic polymorphs at 0 K. In turn, the difference in the total energies of the polymorphs obtained in this way decreased to 1.26 kJ/mol. Formally, the orthorhombic polymorph is more stable; however, the value of energy difference was calculated for 0 K without inclusion of entropy and vibrational terms. According to Nyman and Day [40], at ambient conditions (room temperature), the use of entropy and vibrational terms to calculate differences in energy (in this case, it is not total energy but free energy) lead to re-ranking of the mutual stability in 9% of polymorph pairs. Unfortunately, the calculation of phonon frequencies that is necessary for the evaluation of entropy and vibrational terms at ambient conditions is too time consuming even for small crystals. Although the DFT calculations predict the orthorhombic polymorph to be more stable than the tetragonal one at 0 K, the ranking can change with temperature. The reason for the increase in volume is the weakening of the staking interaction both in tetragonal and orthorhombic polymorphs; however, in the former case, it is more pronounced. At the same time, N-H…O bonds in H-bonded chains are the same as those compared in the cases of experimental single crystal and powder structures.

A series of semiqualitative approaches based on the summation of energies of intermolecular interactions (taken from charge density descriptors, UNI (UNIversal force field) potentials and CE--B3LYP/6-31G(d,p) calculations) were attested to compare the stability of two polymorphs. The charge density study in terms of R. Bader’s “Atoms” in molecules theory [41] was carried out for the structures of polymorphs optimized in experimental cell parameters. According to the charge density study, the bcps corresponding to covalent bond, intramolecular O-H…O bond and intermolecular interactions were revealed. The energies of intra- and intermolecular interactions were estimated with the formula proposed by Espinosa, Mollins and Lecomte for hydrogen bonds [22]. The strength of the intramolecular O-H…O bond in both polymorphs is ~−93.9 kJ/mol (Table 3). The strongest intermolecular N-H-O bond that is responsible for the formation of H-bonded chains is as strong as −37.8 kJ/mol. At the same time, the energy of the N-H…N bond between the amine group and the nitrogen atom of the pyrazine ring is noticeably different in orthorhombic and tetragonal polymorphs (−18.9 and −16.2 kJ/mol). The energies of the rest of the intramolecular interactions are less than −4.2 kJ/mol; however, the total energy of F…O, O…C and N…C interactions that correspond to aromatic stacking is −14.7 kJ/mol. In turn, total energy of all intermolecular interactions in orthorhombic and tetragonal polymorphs is −90.6 and −86.4 kJ/mol. In fact, this value corresponds to the lattice energy and illustrates the mutual stability of the polymorphs. Thus, from a charge density point of view, the orthorhombic polymorph appears to be more stable than the tetragonal one. Thus, total energy values at 0 K and the lattice energy both indicate that the orthorhombic polymorph is more stable. Experimentally, we observed that the ratio of the orthorhombic and tetragonal polymorphs in one sample left for 10 months changes from 0.06:0.94 to 0.15:0.85 (Figure S3), which confirms that the tetragonal polymorph is the metastable form.

Unfortunately, faster and user-friendly calculations based on pairwise interactions failed to rank polymorph stability. Crystal packing energy for the experimentally observed orthorhombic and tetragonal polymorphs estimated using UNI potentials [42,43] is equal to, respectively, −115.5 and −119.3 kJ/mol, and for the optimized crystal structures, the corresponding values are −119.6 and −119.1 kJ/mol. Lattice energies estimated with the method suggested by Mackenzie et al. [44] also resulted in inversed values for the stability of polymorphs (−95.6 and −142.9 kJ/mol for the orthorhombic and tetragonal polymorphs; Table S1). Thus, ranking of polymorph stabilities based on analysis of pairwise interactions should be carefully used. For CE-B3LYP, this may also be indicative of the need to change the basis set [45]. At the same time, application of polymorph ranking based on the EML formula [22] should also be carefully used, as was demonstrated by Spackman [46]. For favipiravir, this approach obtained correct values, as (i) c.a. 3/4 of crystal packing energy corresponded to H-bonds that were analyzed in the original paper [22], and (ii) energies of interactions that corresponded to aromatic stacking appeared to be the same in the two polymorphs.

### 3.4. Hirshfeld Analysis of Two Polymorphs

**Figure 5:**
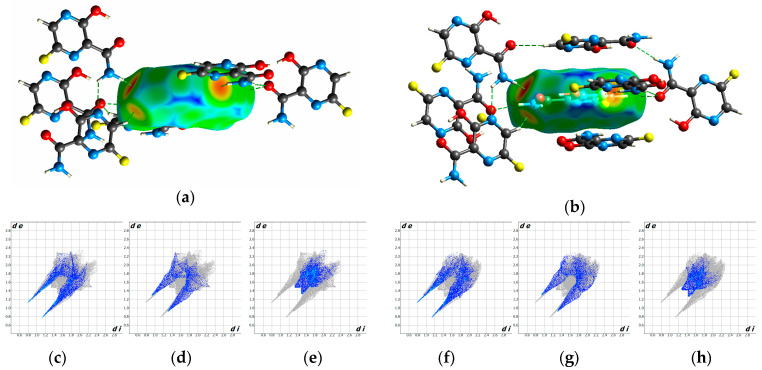
d_e_ plotted on atomic Hirshfeld surface (close contacts are red and elongated are blue) for favipiravir molecule in (**a**) the orthorhomibc polymorph, (**b**) tetragonal polymorph; corresponding fingerprint plots delineated into contribution of O...H, N...H and F...H interactions for the orthorhombic (**c**–**e**) and the tetragonal (**f**–**h**) polymorphs.

Hirshfeld surface analysis of two polymorphs was carried out using Crystal Explorer 17.5 [47], as it was proved to be a valuable tool for the analysis of polymorphs [48,49,50] sensitive to both weak and strong interactions. The molecular Hirshfeld surfaces of favipiravir in two polymorphs are depicted in Figure 5a,b, mapped with d_e_. The closest distances to an external atom (d_e_) enable the visualization of the closest intermolecular interactions colored with the red spots. For both polymorphs, the deep red spots on the Hirshfeld surface represent the O–H···O hydrogen bonds, orange—the N-H...N and yellow—the C-H...O bond. The same information about bond distances can be derived from the two-dimensional fingerprint plots given in Figure 5c–h, where the H...O and N...H bonds show the closest intermolecular interactions and form sharp peaks on the plots. Distributions for the H...F interactions are more diffused, and these are situated at longer distances. A summary of the relative contributions of intermolecular contacts in the area of molecular surfaces is given in Table 4. Contribution of the O...H, N...H, H...H and F...F interactions to the surface is lower for the orthorhombic polymorph (18.2, 11.2, 12.7 and 3.7% as compared with 19.0, 12.9, 13.6 and 5.3%), but is larger for the F...H, C...H and N...C interactions (14.9, 9.5 and 8.1% as compared with 11.2, 8.1 and 6.0%). Contributions of the other interactions are nearly the same and do not exceed 4.6%. All 15 theoretically possible types of interactions are observed in the tetragonal polymorph, while C...C and O...O interactions are absent in the orthorhombic polymorph.

Finally, we compared the contributions of various types of intermolecular interactions to the Hirshfeld surface (C_XY_) with their theoretical proportion of random contacts R_XY_ as described in [51]. The enrichment ratios E_XY_ = C_XY_/R_XY_ are given in Table 4, and the values >>1.0 are marked in bold to denote pairs of elements that tend to form interactions. E_XY_ << 1 are associated with atomic pairs that are inclined to avoid contacts. For both polymorphs, the interactions with high propensity to appear are the O..H and F...H hydrogen bonds and F...F and F...O bonds. Among them, F...F, F...O and F...H interactions appear much more often than it was expected by chance, while F...N and F...C interactions are disfavored. Indeed, for the tetragonal polymorph, bond critical points were found for F1...F1, F1...O1, F1...N3 and C-H...F1 interactions with the energy sum of −13.1 kJ/mol, as obtained from the EML correlation [33]. In the orthorhombic polymorph, fluorine atom takes part in interactions with the energy sum of −12.9 kJ/mol.

### 3.5. Analysis of H-Bonding Network

**Figure 6:**
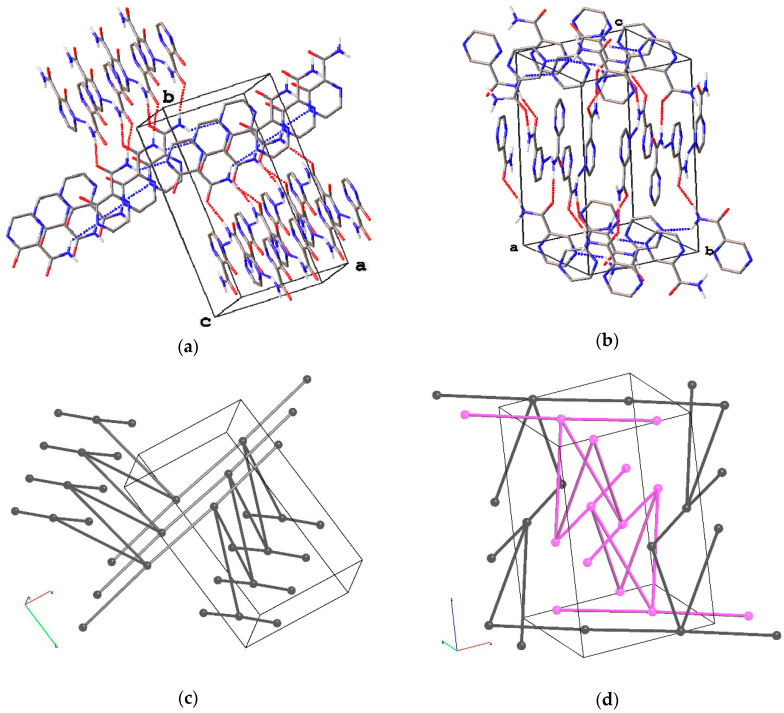
Fragment of H-bonded networks in (**a**) orthorhombic and (**b**) tetragonal polymorphs of favipiravir and their underlying nets ((**c**) **zst** and (**d**) 4/4/t39, respectively). H(C) and F atoms are omitted. Two interpenetrated networks are depicted with black and fuchsia.

The difference in mutual position of favipiravir molecules connected through the strong H-bonds results in prominent variation of the overall packing of the two polymorphs. The underlying H-bonded network for the orthorhombic polymorph realizes the three-periodic four-connected (4c) **zst** topology (Figure 6a,c), as described in [52]. In the tetragonal polymorph, favipiravir molecules form two interpenetrated three-periodic networks each with the 4/4/t39 topology (or [4.10^16^.6^2^.6^2^.6^2^.6^2^]; Figure 6c,d). Both H-bonding networks are three-periodic, and their energies as we mentioned above are roughly comparable; thus, one can hardly expect any difference in their mechanical properties. The **zst** and 4/4/t39 topologies for H-bonded networks were previously met in 2 and 14 crystal structures of small molecules in accord with the Topcryst resource [53]. It is worth mentioning that favipiravir, orcinol {EWAMAR01} [54] and methylimino-diacetic acid {FENTOH02} [55] with the 4/4/t39 topology of the underlying net form an orthorhombic and an orthogonal polymorph, although topologies of H-bonding architectures in orthorhombic polymorphs do not coincide for the three different compounds. Particularly, for methylimino-diacetic acid that also has two interpenetrated H-bonded nets with the 4/4/t39 topology in the tetragonal polymorph, the orthorhombic polymorph contains a two-periodic H-bonded net with **sql** (square lattice) topology. Overall, the observed polymorphism of favipiravir demonstrated a very rare example of polymorphism associated with different topologies of H-bonded architectures, one of which had two interpenetrated architectures. Particularly for small organic molecules taken from the Cambridge Structural Database in 2006, only seven polymorph families were found, with at least one polymorph realizing H-bonded interpenetrated architectures [56].

DSC measurements of both samples indicate no phase transitions in the solid state at heating, although powder XRD data of the fresh sample and the sample after 10 months demonstrated partial conversion of the metastable tetragonal polymorph to the orthorhombic one. Thus, additional analysis of unit cells and net relations was carried out. The orthorhombic phase has unit cell parameters similar to those of the tetragonal phase. However, neither Pna2_1_ space group, nor any of its supergroups are among the subgroups of the P4_2_/n space group. Thus, direct phase transition between two polymorphs is forbidden. Recently, the network topological model of solid-state transformations was suggested [57,58]. The method consists of screening the subnets and/or supernets which can represent the potential connectivities of the intermediate structures or the resulting stable phases in the process of solid-state transformation. Analysis of net relations between experimentally observed **zst** and two interpenetrated 4/4/t39 nets was carried out using ToposPro software [59]. This also revealed no common subnets and supernets for these architectures (Figure S4), as analyzed within the experimental unit cells. This may explain why the metastable phase very slowly transforms to the stable one.

## 4. Conclusions

A novel polymorph of favipiravir was obtained and characterized using FT-IR spectra and DSC measurements. Its crystal structure was determined using powder X-ray diffraction as the tetragonal polymorph with crystal parameters similar with those of the known orthorhombic polymorph. The orthorhombic polymorph is more readily obtained from different solvents and realizes a more widespread H-bonding architecture. The tetragonal polymorph slightly converts to the orthorhombic one at room temperature. The periodic DFT calculations with Grimme’s D3 correction and lattice energy calculations both indicate that the tetragonal polymorph is metastable. The lattice energy calculation with UNI potentials and CE-B3LYP model energies based on experimental data failed to predict the most stable polymorph, which indicates that such approximate calculations should be carefully used. Analysis of intermolecular interactions indicates the similarity in the geometry and energy of hydrogen bonds.

## Figures and Tables

**Table 1:** Summary of the powder patterns depicted in Figure 1 refinement.

Unit Cell Parameters	Tetragonal *	Orthorhombic **
a, Å	9.2805(7)	9.1133(6)
b, Å	9.2805(7)	14.7708(10)
c, Å	14.6491(13)	4.6893(3)
V, Å^3^	1261.7(2)	631.23(7)
ρ, g/cm^3^	1.6557(3)	1.6531(2)
R_wp_, %	5.190	8.339
R_bragg_, %	3.394	4.537

* The sample depicted in Figure 1a contains 6.4(4)% of the orthorhombic modification. ** Powder pattern is depicted in Figure 1b.

**Table 2:** Propensities of H-bonding in two polymorphs of favipiravir.

Donor	Acceptor	Propensity	Observed in Orthorhombic Polymorphs	Observed in Tetragonal Polymorphs
**Intermolecular**				
NH (amide)	N3 (pyrazine)	0.78	Yes	Yes
NH (amide)	O1 (amide)	0.77	Yes	Yes
NH (amide)	N2 (pyrazine)	0.75		
OH	N3(pyrazine)	0.50		
OH	O1 (amide)	0.48		
OH	N2 (pyrazine)	0.46		
NH (amide)	OH	0.42		
OH	OH	0.17		
**Intramolecular**				
NH (amide)	OH	0.88		
OH	O1 (amide)	0.84	Yes	Yes
NH (amide)	N2 (pyrazine)	0.62		

**Table 3:** Hydrogen bonds for optimized polymorphs of favipiravir.

D-H...A ^a^	d(D-H), Å	d(H...A), Å	d(D...A), Å	<(DHA), °	ρ(r) ^b^, a.u.	∇^2^ρ(r) ^b^, a.u.	E_int_^b^, kJ/mol
**Orthorhombic Polymorph**							
O2-H1...O1	1.017	1.602	2.545	152	0.064	0.142	−93.9
N1-H3...O1#1	1.030	1.817	2.836	169	0.035	0.084	−37.8
N1-H2...N3#2	1.024	2.144	2.981	138	0.021	0.062	−18.9
C4-H4...O1#3	1.092	2.386	3.391	152	0.010	0.040	−8.1
C4-H4...F1#4	1.092	2.861	3.447	114	0.001	0.008	−1.0
**Tetragonal Polymorph**							
O2-H1...O1	1.017	1.599	2.543	152	0.064	0.144	−93.9
N1-H3...O1#5	1.029	1.820	2.839	170	0.035	0.083	−37.2
N1-H2...N3#6	1.023	2.202	3.018	135	0.019	0.058	−16.2
C4-H4...O1#7	1.091	2.406	3.381	148	0.010	0.039	−7.8
C4-H4...F1#8	1.091	2.795	3.346	111	0.004	0.019	−2.8

^a^ Symmetry transformations used to generate equivalent atoms: #1 1−x, 1−y, −1/2+z; #2 −1/2+x, 1/2−y, −1+z; #3 3/2−x, −1/2+y, 1/2+z; #4 1−x, −y, 1/2+z; #5 y, −1/2−x, 1/2−z; #6 1/2+x, −1/2+y,−z; #7 −1/2+y, −x, −1/2+z; #8 −1/2−y, x, −1/2−z. ^b^ ρ(**r**)—electron density at bcps; ∇^2^ρ(**r**)—Laplacian of electron density at bcps; E_int_—energy of an interaction estimated using the correlation proposed in [22] as −0.5V(**r**).

**Table 4:** Contributions C_XY_ of various types of intermolecular interactions to the molecular Hirshfeld surface of two polymorphs, their R_XY_ values and enrichment ratios E_XY_.

Interaction	R_XY_, %	C_XY_, ^a^ %		E_XY_	
		Orthorhombic	Tetragonal	Orthorhombic	Tetragonal
O...H	7.1	18.2	19.0	**2.6**	**2.7**
N...H	10.7	11.2	12.9	1.1	1.2
F...H	3.6	14.9	11.2	**4.2**	**3.2**
C..H	17.8	9.5	8.1	0.5	0.5
C...C	11.1	0.0	1.7	0.0	0.2
H...H	7.1	12.7	13.6	1.8	1.9
O...O	1.8	0.0	0.3	0.0	0.2
O...N	5.3	4.6	4.3	0.9	0.8
O...C	8.9	7.0	7.4	0.8	0.8
N...C	13.3	8.1	6.0	0.6	0.5
N...N	4.0	3.8	4.1	1.0	1.0
F...F	0.4	3.7	5.3	**8.3**	**11.9**
F...O	1.8	4.5	2.9	**2.5**	1.6
F...N	2.7	0.2	1.3	0.1	0.5
F...C	4.4	1.4	1.9	0.3	0.4

^a^ Atomic coordinates and unit cell parameters for experimentally obtained crystals structures were taken.

## Data Availability

The data presented in this study are available in supplementary materials.

## Supplementary Materials

The following are available online at https://www.mdpi.com/1999-4923/13/2/139/s1, Figure S1: IR spectra of two polymorphs; Figure S2: DSC data for two polymorphs; Figure S3: XRD patterns of favipiravir recrystallized from ethyl acetate after 10 months; Figure S4: topological analysis of H-bonding architectures in two polymorphs; atomic coordinates for optimized structures; Table S1. Energies (kJ mol^−1^) of intermolecular interactions in favipiravir polymorphs calculated by the UNI force field and the dimer interaction energies calculated using Crystal Explorer based on the PIXEL method [CE-B3LYP/6-31G(d,p)]; Supplementary material S1. The results of DFT calculations.
