# Supplementary material for: Novel Polymorph of Favipiravir—An Antiviral Medication
Source: Pharmaceutics. 2021 Jan 21;13(2):139. doi: 10.3390/pharmaceutics13020139 (PMC7911870; doi:10.3390/pharmaceutics13020139)
Supplement: Supplementary file 1 [file pharmaceutics-13-00139-s001.pdf]

pharmaceutics

MDPI

# Supplementary Materials: Novel Polymorph of Favipiravir—an Antiviral Medication

**Alexander Goloveshkin, Alexander A. Korlyukov and Anna V. Vologzhanina**

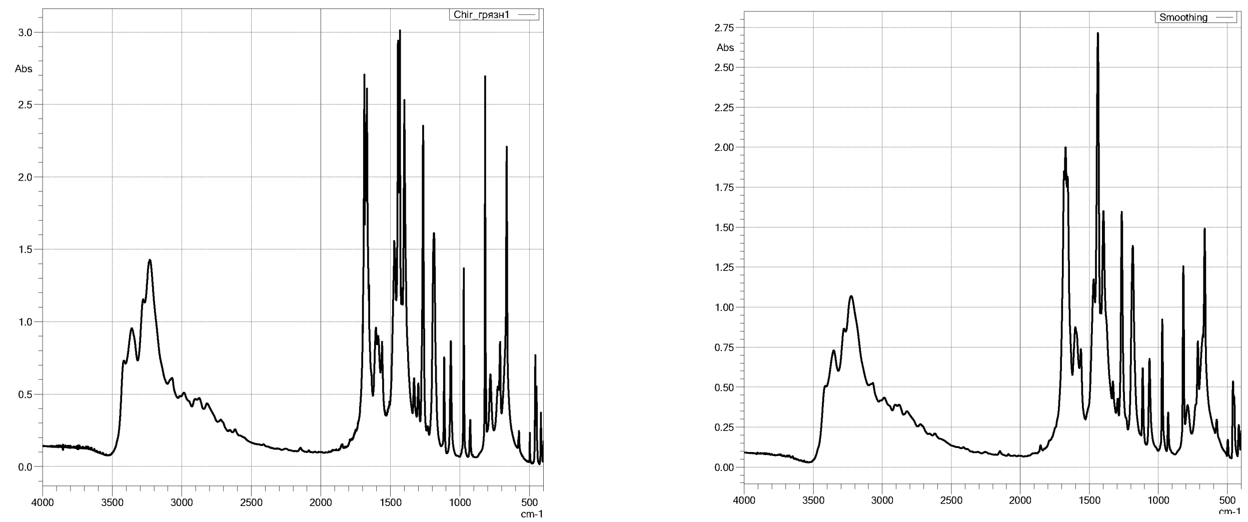


(**a**) (**b**)

**Figure S1.** FT-IR spectra of the **(a)** tetragonal and **(b)** orthorhombic polymorphs of favipiravir.

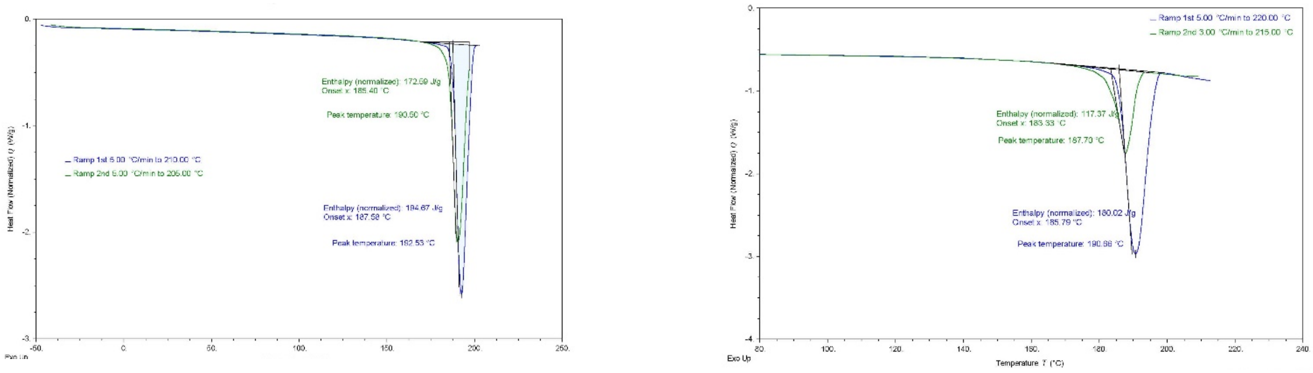


(**a**) (**b**)

**Figure S2.** DSC curves of the **(a)** tetragonal and **(b)** orthorhombic polymorphs of favipiravir.

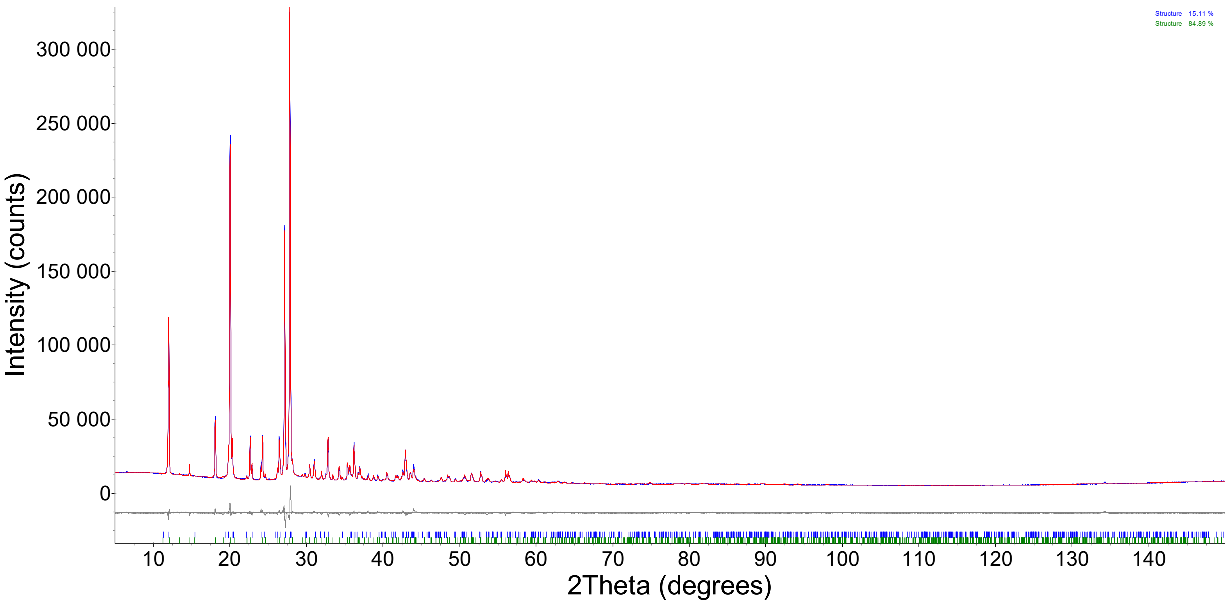


**Figure S3.** Powder XRD patterns of favipiravir recrystallized from ethyl acetate after 10 months of staying. The Rietveld refinement gives ration of the tetragonal and orthorhombic forms as 0.85 : 0.15. Experimental (blue), calculated (red) and their difference (grey) curves are depicted.

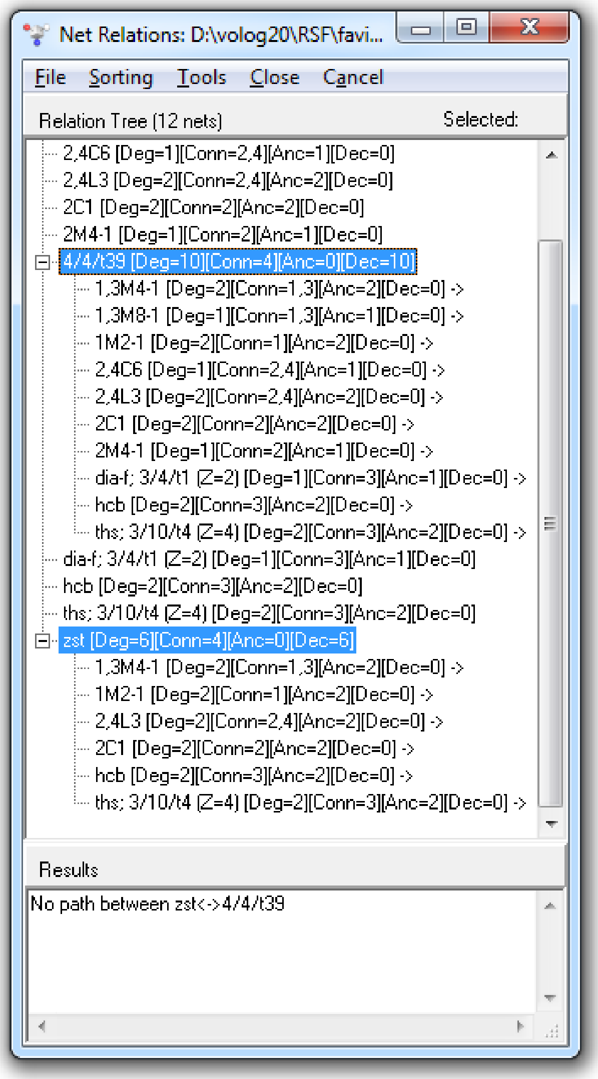


**Figure S4.** Net relations between **zst** and 4/4/t39 nets.

**Table S1.** Energies (kJ mol$^{-1}$) of intermolecular interactions in favipiravir polymorphs calculated by the UNI force field and the dimer interaction energies calculated using *Crystal Explorer* based on the PIXEL method [CE–B3LYP/6–31 G(d,p)].

| Symmetry Operation; Multiplicity | UNI Empirical Force Field | CE–B3LYP/6–31G(d,p) | | | | |
|---|---|---|---|---|---|---|
| | | $E_{el}$ | $E_{pol}$ | $E_{dis}$ | $E_{rep}$ | $E_{tot}$ |
| Orthorhombic polymorph | | | | | | |
| −x+1/2, y+1/2, z+1/2; 2 | −5.9 | −5.6 | −1.5 | −5.6 | 7.2 | −7.5 |
| x+1/2, −y+1/2, z; 2 | −14.4 | −2.5 | −1.5 | −23 | 11 | −17.1 |
| x+1/2, −y+1/2, z; 2 | −28.2 | −18.8 | −6.7 | −11.5 | 25.1 | −19.3 |
| −x, −y, z+1/2; 2 | −2.6 | −0.8 | −0.1 | −4.7 | 1.3 | −4.3 |
| −x+1/2, y+1/2, z+1/2; 2 | −5.1 | −1.2 | −0.2 | −3.8 | 0.2 | −4.6 |
| x, y, z; 2 | −18.1 | −3.1 | −1.6 | −21.5 | 8.6 | −17.8 |
| −x, −y, z+1/2; 2 | −28.6 | −35.9 | −7.8 | −7,7 | 41.2 | −25 |
| Tetragonal polymorph | | | | | | |
| x+1/2, y+1/2, −z; 2 | −18 | −17 | −6.1 | −11,2 | 21 | −19.2 |
| y+1/2, −x, z+1/2; 2 | −5.7 | −1.2 | −0.2 | −4 | 0.3 | −4.8 |
| y+1/2, −x, z+1/2; 2 | −6.2 | −4.8 | −1.5 | −5.9 | 7 | −7 |
| −x, −y, −z; 1 | −31 | −11 | −2.1 | −24 | 11.8 | −26.8 |
| −x, −y, −z; 1 | −23.4 | 1.4 | −1.1 | −21.3 | 8.7 | −12.5 |
| −x+1/2, −y+1/2, z; 1 | −31 | −8.7 | −2.3 | −25.2 | 12.7 | −25 |
| y, −x+1/2, −z+1/2; 2 | −2.5 | −1 | −0.2 | −5.4 | 2 | −4.7 |
| −x+1/2, −y+1/2, z; 1 | −24.4 | −4.4 | −1 | −18.5 | 5.8 | −17.9 |
| y, −x+1/2, −z+1/2; 2 | −14.3 | −35.7 | −7.7 | −7.6 | 40.6 | −25 |

**Supplementary material S1.** The results of DFT calculations

The geometry of orthorhombic phase of favipiravir optimized in experimental parameters (VASP POSCAR format)

```
C5 H4 F N3 O2
   1.00000000000000
     9.1106004714999997    0.0000000000000000    0.0000000000000000
     0.0000000000000000   14.7618999480999999    0.0000000000000000
     0.0000000000000000    0.0000000000000000    4.6909999847000003
   F    O    N    C    H
     4    8   12   20   16
Direct
  0.5289410000000032  0.0630150000000000  0.3958650000000006
  0.4710589999999968  0.9369850000000000  0.8958650000000006
  0.0289410000000032  0.4369850000000000  0.3958650000000006
  0.9710589999999968  0.5630150000000000  0.8958650000000006
  0.6447379999999967  0.4448539999999994  0.6018830000000008
  0.3552620000000033  0.5551460000000006  0.1018830000000008
  0.1447379999999967  0.0551460000000006  0.6018830000000008
  0.8552620000000033  0.9448539999999994  0.1018830000000008
  0.7974740000000011  0.3393769999999989  0.9118479999999991
  0.2025259999999989  0.6606230000000011  0.4118479999999991
  0.2974740000000011  0.1606230000000011  0.9118479999999991
  0.7025259999999989  0.8393769999999989  0.4118479999999991
  0.4806639999999973  0.3886419999999973  0.2798719999999975
  0.5193360000000027  0.6113580000000027  0.7798719999999975
  0.9806639999999973  0.1113580000000027  0.2798719999999975
  0.0193360000000027  0.8886419999999973  0.7798719999999975
  0.5559719999999970  0.2150940000000006  0.4394909999999967
  0.4440280000000030  0.7849059999999994  0.9394920000000013
  0.0559719999999970  0.2849059999999994  0.4394909999999967
```

0.9440280000000030 0.7150940000000006 0.9394920000000013
0.7637009999999975 0.1871730000000014 0.8682700000000025
0.2362990000000025 0.8128269999999986 0.3682700000000025
0.2637009999999975 0.3128269999999986 0.8682700000000025
0.7362990000000025 0.6871730000000014 0.3682700000000025
0.5819759999999974 0.3784029999999987 0.4801459999999977
0.4180240000000026 0.6215970000000013 0.9801459999999977
0.0819759999999974 0.1215970000000013 0.4801459999999977
0.9180240000000026 0.8784029999999987 0.9801459999999977
0.6223460000000003 0.2852329999999981 0.5671430000000015
0.3776539999999997 0.7147670000000019 0.0671430000000015
0.1223460000000003 0.2147670000000019 0.5671430000000015
0.8776539999999997 0.7852329999999981 0.0671430000000015
0.7285370000000029 0.2712450000000004 0.7836879999999979
0.2714629999999971 0.7287549999999996 0.2836879999999979
0.2285370000000029 0.2287549999999996 0.7836879999999979
0.7714629999999971 0.7712450000000004 0.2836879999999979
0.6962490000000017 0.1180730000000025 0.7414020000000008
0.3037509999999983 0.8819269999999975 0.2414020000000008
0.1962490000000017 0.3819269999999975 0.7414020000000008
0.8037509999999983 0.6180730000000025 0.2414020000000008
0.5933940000000035 0.1341469999999987 0.5256040000000013
0.4066059999999965 0.8658530000000013 0.0256040000000013
0.0933940000000035 0.3658530000000013 0.5256040000000013
0.9066059999999965 0.6341469999999987 0.0256040000000013
0.7230100000000022 0.0494729999999990 0.8113880000000009
0.2769899999999978 0.9505270000000010 0.3113880000000009
0.2230100000000022 0.4505270000000010 0.8113880000000009
0.7769899999999978 0.5494729999999990 0.3113880000000009
0.4474149999999995 0.4522660000000016 0.2168920000000014
0.5525850000000005 0.5477339999999984 0.7168920000000014
0.9474149999999995 0.0477339999999984 0.2168920000000014
0.0525850000000005 0.9522660000000016 0.7168920000000014
0.4295770000000019 0.3346779999999967 0.1850310000000022
0.5704229999999981 0.6653220000000033 0.6850310000000022
0.9295770000000019 0.1653220000000033 0.1850310000000022
0.0704229999999981 0.8346779999999967 0.6850310000000022
0.7549679999999981 0.3957169999999977 0.8184770000000015
0.2450320000000019 0.6042819999999978 0.3184770000000015
0.2549679999999981 0.1042830000000023 0.8184770000000015
0.7450320000000019 0.8957180000000022 0.3184770000000015
Total energy = −412.81351961 eV

The geometry of orthorhombic phase of favipiravir entirely optimized (VASP POSCAR format)

C5 H4 F N3 O2
1.00000000000000
9.4997554558584323 0.0000000000000000 0.0000000000000000
0.0000000000000000 15.0155879696109640 0.0000000000000000
0.0000000000000000 0.0000000000000000 4.8536951841730431
C H F N O
20 16 4 12 8
Direct
0.5762005835212705 0.3782561461902461 0.4761132017032779

| | | |
|---|---|---|
| 0.4237994164787295 | 0.6217438238097515 | 0.9761131717032754 |
| 0.0762005835212705 | 0.1217438538097539 | 0.4761132017032779 |
| 0.9237994164787295 | 0.8782561761902485 | 0.9761131717032754 |
| 0.6176026155359295 | 0.2875539120208117 | 0.5677093878156754 |
| 0.3823973844640776 | 0.7124460579791858 | 0.0677093878156754 |
| 0.1176026155359224 | 0.2124460879791883 | 0.5677093878156754 |
| 0.8823973844640705 | 0.7875539420208142 | 0.0677093878156754 |
| 0.7214725804142930 | 0.2762520825115189 | 0.7752669286099518 |
| 0.2785274195857070 | 0.7237479174884811 | 0.2752669286099518 |
| 0.2214725804142930 | 0.2237479174884811 | 0.7752669286099518 |
| 0.7785274195857070 | 0.7762520825115189 | 0.2752669286099518 |
| 0.6937635117245193 | 0.1251757090265997 | 0.7469428281472261 |
| 0.3062364882754736 | 0.8748243059733980 | 0.2469428281472261 |
| 0.1937635117245193 | 0.3748243059733980 | 0.7469428281472261 |
| 0.8062364882754807 | 0.6251756940266020 | 0.2469428281472261 |
| 0.5928336796411600 | 0.1383272654540875 | 0.5403654732873520 |
| 0.4071663203588400 | 0.8616727345459125 | 0.0403654732873520 |
| 0.0928336796411600 | 0.3616727345459125 | 0.5403654732873520 |
| 0.9071663203588400 | 0.6383272654540875 | 0.0403654732873520 |
| 0.7215813134602129 | 0.0583604359200010 | 0.8176050089676679 |
| 0.2784186865397871 | 0.9416395790799967 | 0.3176050089676679 |
| 0.2215813134602129 | 0.4416395790800038 | 0.8176050089676679 |
| 0.7784186865397871 | 0.5583604209200033 | 0.3176050089676679 |
| 0.4476261377627893 | 0.4467877598214898 | 0.2107216955605011 |
| 0.5523738322372083 | 0.5532122101785077 | 0.7107217255605036 |
| 0.9476261677627917 | 0.0532122401785102 | 0.2107216955605011 |
| 0.0523738622372107 | 0.9467877898214923 | 0.7107217255605036 |
| 0.4326315495357207 | 0.3302550981742698 | 0.1950250177079198 |
| 0.5673684504642793 | 0.6697449018257302 | 0.6950250027079221 |
| 0.9326315495357207 | 0.1697449018257302 | 0.1950250177079198 |
| 0.0673684504642793 | 0.8302550981742698 | 0.6950250027079221 |
| 0.7441413505156405 | 0.3991576585825740 | 0.7994319077557464 |
| 0.2558586494843595 | 0.6008423114174306 | 0.2994319077557464 |
| 0.2441413505156405 | 0.1008423414174260 | 0.7994319077557464 |
| 0.7558586494843595 | 0.8991576885825694 | 0.2994319077557464 |
| 0.5313130059907536 | 0.0668563336817911 | 0.4230280832564546 |
| 0.4686869940092464 | 0.9331436513182112 | 0.9230280532564521 |
| 0.0313130059907536 | 0.4331436513182112 | 0.4230280832564546 |
| 0.9686869940092464 | 0.5668563486817888 | 0.9230280532564521 |
| 0.4785637506297817 | 0.3852088801538471 | 0.2806770276123558 |
| 0.5214362493702183 | 0.6147911198461458 | 0.7806770276123558 |
| 0.9785637506297817 | 0.1147911198461529 | 0.2806770276123558 |
| 0.0214362493702183 | 0.8852088801538542 | 0.7806770276123558 |
| 0.5546175238429853 | 0.2169158615412456 | 0.4517582802846292 |
| 0.4453824761570147 | 0.7830841534587520 | 0.9517582802846292 |
| 0.0546175238429853 | 0.2830841534587520 | 0.4517582802846292 |
| 0.9453824761570147 | 0.7169158465412480 | 0.9517582802846292 |
| 0.7580452265561064 | 0.1945462629611683 | 0.8626556784378963 |
| 0.2419547734438936 | 0.8054537370388317 | 0.3626556784378963 |
| 0.2580452265561064 | 0.3054537370388317 | 0.8626556784378963 |
| 0.7419547734438936 | 0.6945462629611683 | 0.3626556784378963 |
| 0.6342143627384829 | 0.4452666925399029 | 0.5875784174459397 |
| 0.3657856372615171 | 0.5547333074600971 | 0.0875784174459397 |
| 0.1342143627384829 | 0.0547333074600900 | 0.5875784174459397 |

```
0.8657856372615171 0.9452666925399029 0.0875784174459397
0.7867763704692905 0.3449245192942030 0.8921210214073767
0.2132236295307095 0.6550754807057970 0.3921210214073767
0.2867763704692976 0.1550754807057970 0.8921210214073767
0.7132236295307095 0.8449245192942030 0.3921210214073767
```

Total energy = −412.90789928 eV

The geometry of tetragonal phase of favipiravir optimized in experimental parameters (VASP POSCAR format)

```
C40 H32 F8 N24 O16
  1.00000000000000
    9.6594038995192619  −0.0000000052983579  −0.0000000000000000
    0.0000000052983579   9.6594038995192619   0.0000000000000000
   −0.0000000000000000   0.0000000000000000  14.8975776383443037
  F  O  H  N  C
  8  16  32  24  40
Direct
0.3427963850371691 0.0872801151780781 0.1870605134702803
0.6572035859628320 0.9127198848219216 0.8129394565297239
0.1572036149628309 0.4127198848219149 0.1870605134702803
0.8427964140371680 0.5872801151780784 0.8129394565297239
0.9127198848219216 0.8427964140371680 0.6870605434702761
0.0872801151780781 0.1572036149628309 0.3129394865297201
0.5872801151780784 0.6572035859628320 0.6870605434702761
0.4127198848219149 0.3427963850371691 0.3129394865297201
0.2434405112382017 0.9935084562717965 0.8064571446732783
0.7565594887617986 0.0064915437282030 0.1935428553267215
0.2565594887617987 0.5064915437282035 0.8064571446732783
0.7434405112382014 0.4935084562717974 0.1935428553267215
0.0064915437282030 0.7434405112382014 0.3064571446732786
0.9935084562717965 0.2565594887617987 0.6935428553267217
0.4935084562717974 0.7565594887617986 0.3064571446732786
0.5064915437282035 0.2434405112382017 0.6935428553267217
0.0947569538290090 0.8421332741541537 0.9089562180372622
0.9052430611709892 0.1578667408458516 0.0910437969627426
0.4052430611709888 0.6578667258458463 0.9089562180372622
0.5947569388290108 0.3421332741541532 0.0910437969627426
0.1578667408458516 0.5947569388290108 0.4089562180372622
0.8421332741541537 0.4052430611709888 0.5910437819627378
0.3421332741541532 0.9052430611709892 0.4089562180372622
0.6578667258458463 0.0947569538290090 0.5910437819627378
0.1383671439214208 0.8860638766708747 0.8538716416539953
0.8616328490785820 0.1139361083291277 0.1461283283460020
0.3616328490785822 0.6139361233291253 0.8538716416539953
0.6383671509214180 0.3860638766708747 0.1461283283460020
0.1139361083291277 0.6383671509214180 0.3538716716539981
0.8860638766708747 0.3616328490785822 0.6461283583460047
0.3860638766708747 0.8616328490785820 0.3538716716539981
0.6139361233291253 0.1383671439214208 0.6461283583460047
0.4299637024884405 0.1804305636387983 0.8030628718144213
0.5700362975115664 0.8195694363612017 0.1969371431855769
0.0700362975115596 0.3195694363612018 0.8030628718144213
0.9299637024884336 0.6804305636387983 0.1969371431855769
0.8195694363612017 0.9299637024884336 0.3030628718144209
0.1804305636387983 0.0700362975115596 0.6969371281855787
```

| | | |
|---|---|---|
| 0.6804305636387983 | 0.5700362975115664 | 0.3030628718144209 |
| 0.3195694363612018 | 0.4299637024884405 | 0.6969371281855787 |
| 0.4438592044252098 | 0.1905318100678917 | 0.9205528473687543 |
| 0.5561407955747901 | 0.8094681599321056 | 0.0794471376312551 |
| 0.0561407955747902 | 0.3094681899321079 | 0.9205528473687543 |
| 0.9438592044252099 | 0.6905318400678944 | 0.0794471376312551 |
| 0.8094681599321056 | 0.9438592044252099 | 0.4205528473687474 |
| 0.1905318100678917 | 0.0561407955747902 | 0.5794471526312457 |
| 0.6905318400678944 | 0.5561407955747901 | 0.4205528473687474 |
| 0.3094681899321079 | 0.4438592044252098 | 0.5794471526312457 |
| 0.1438499820601694 | 0.9006911693039169 | 0.1974072518130192 |
| 0.8561500329398354 | 0.0993088156960856 | 0.8025927331869835 |
| 0.3561500329398355 | 0.5993088306960831 | 0.1974072518130192 |
| 0.6438499670601646 | 0.4006911693039164 | 0.8025927331869835 |
| 0.0993088156960856 | 0.6438499670601646 | 0.6974072668130165 |
| 0.9006911693039169 | 0.3561500329398355 | 0.3025927331869835 |
| 0.4006911693039164 | 0.8561500329398354 | 0.6974072668130165 |
| 0.5993088306960831 | 0.1438499820601694 | 0.3025927331869835 |
| 0.3992465220819215 | 0.1465710377226559 | 0.8653678728495585 |
| 0.6007534779180785 | 0.8534289322773413 | 0.1346321421504393 |
| 0.1007534779180788 | 0.3534289622773372 | 0.8653678728495585 |
| 0.8992465220819215 | 0.6465710677226587 | 0.1346321421504393 |
| 0.8534289322773413 | 0.8992465220819215 | 0.3653678728495583 |
| 0.1465710377226559 | 0.1007534779180788 | 0.6346321271504415 |
| 0.6465710677226587 | 0.6007534779180785 | 0.3653678728495583 |
| 0.3534289622773372 | 0.3992465220819215 | 0.6346321271504415 |
| 0.3221068428546564 | 0.0673217272865314 | 0.0359040727507765 |
| 0.6778931271453489 | 0.9326782457134628 | 0.9640959232492226 |
| 0.1778931571453436 | 0.4326782757134655 | 0.0359040727507765 |
| 0.8221068728546511 | 0.5673217542865372 | 0.9640959232492226 |
| 0.9326782457134628 | 0.8221068728546511 | 0.5359040767507774 |
| 0.0673217272865314 | 0.1778931571453436 | 0.4640959232492232 |
| 0.5673217542865372 | 0.6778931271453489 | 0.5359040767507774 |
| 0.4326782757134655 | 0.3221068428546564 | 0.4640959232492232 |
| 0.1154732473067152 | 0.8679045021273228 | 0.0603663236544768 |
| 0.8845267226932904 | 0.1320954828726721 | 0.9396336763455229 |
| 0.3845267526932845 | 0.6320954978726772 | 0.0603663236544768 |
| 0.6154732773067096 | 0.3679045021273231 | 0.9396336763455229 |
| 0.1320954828726721 | 0.6154732773067096 | 0.5603663236544771 |
| 0.8679045021273228 | 0.3845267526932845 | 0.4396336763455233 |
| 0.3679045021273231 | 0.8845267226932904 | 0.5603663236544771 |
| 0.6320954978726772 | 0.1154732473067152 | 0.4396336763455233 |
| 0.3023037453060334 | 0.0496461628511990 | 0.8733412793069996 |
| 0.6976962546939667 | 0.9503538351488073 | 0.1266587356929982 |
| 0.1976962546939666 | 0.4503538351488010 | 0.8733412793069996 |
| 0.8023037453060333 | 0.5496461648511927 | 0.1266587356929982 |
| 0.9503538351488073 | 0.8023037453060333 | 0.3733412793069997 |
| 0.0496461628511990 | 0.1976962546939666 | 0.6266587206930004 |
| 0.5496461648511927 | 0.6976962546939667 | 0.3733412793069997 |
| 0.4503538351488011 | 0.3023037453060334 | 0.6266587206930004 |
| 0.2606765311856996 | 0.0069418645800278 | 0.9653039132276976 |
| 0.7393234388143053 | 0.9930581054199624 | 0.0346960607723076 |
| 0.2393234688143009 | 0.4930581354199720 | 0.9653039132276976 |
| 0.7606765611856947 | 0.5069418945800376 | 0.0346960607723076 |

0.9930581054199695 0.7606765611856947 0.4653039432276931
0.0069418645800278 0.2393234688143009 0.5346960867723024
0.5069418945800376 0.7393234388143053 0.4653039432276931
0.4930581354199720 0.2606765311856996 0.5346960867723024
0.1564195434687055 0.9050688944775320 0.9776597022247269
0.8435804265312915 0.0949311055224677 0.0223402777752782
0.3435804565312950 0.5949311055224680 0.9776597022247269
0.6564195734687085 0.4050688944775321 0.0223402777752782
0.0949311055224677 0.6564195734687085 0.4776597322247226
0.9050688944775320 0.3435804565312950 0.5223402977752731
0.4050688944775321 0.8435804265312915 0.4776597322247226
0.5949311055224680 0.1564195434687055 0.5223402977752731
0.1768289195797618 0.9294672256492088 0.1297710514869487
0.8231710654202409 0.0705327893507886 0.8702289335130468
0.3231710654202409 0.5705327743507912 0.1297710514869487
0.6768289345797591 0.4294672256492091 0.8702289335130468
0.0705327893507886 0.6768289345797591 0.6297710664869532
0.9294672256492088 0.3231710654202409 0.3702289335130468
0.4294672256492091 0.8231710654202409 0.6297710664869532
0.5705327743507912 0.1768289195797618 0.3702289335130468
0.2807070652258103 0.0284220396406243 0.1156352278278773
0.7192929347741823 0.9715779543593747 0.8843647571721249
0.2192929347741895 0.4715779543593754 0.1156352278278773
0.7807070652258177 0.5284220456406253 0.8843647571721249
0.9715779543593747 0.7807070652258177 0.6156352428278751
0.0284220396406243 0.2192929347741895 0.38436f47571721245
0.5284220456406253 0.7192929347741823 0.6156352428278751
0.4715779543593754 0.2807070652258103 0.3843647571721245

Total energy = −825.25388750 eV

The geometry of orthorhombic phase of favipiravir entirely optimized (VASP POSCAR format)

C40 H32 F8 N24 O16
1.00000000000000
9.2751998900999997 0.0000000000000000 0.0000000000000000
0.0000000000000000 9.2751998900999997 0.0000000000000000
0.0000000000000000 0.0000000000000000 14.6443004608000003
F O H N C
8 16 32 24 40
Direct
0.3489697395244922 0.0983232435047875 0.1893327043003330
0.6510302314755096 0.9016767564952123 0.8106672656996718
0.1510302604755080 0.4016767564952127 0.1893327043003330
0.8489697685244904 0.5983232435047877 0.8106672656996718
0.9016767564952123 0.8489697685244904 0.6893327343003282
0.0983232435047875 0.1510302604755080 0.3106672956996669
0.5983232435047877 0.6510302314755096 0.6893327343003282
0.4016767564952127 0.3489697395244922 0.3106672956996669
0.2373337871072032 0.9782326021543593 0.8062376518831177
0.7626662128927976 0.0217673978456406 0.1937623481168824
0.2626662128927969 0.5217673978456407 0.8062376518831177
0.7373337871072024 0.4782326021543594 0.1937623481168824
0.0217673978456406 0.7373337871072024 0.3062376518831177
0.9782326021543593 0.2626662128927969 0.6937623481168823
0.4782326021543594 0.7626662128927976 0.3062376518831177

| | | |
|---|---|---|
| 0.5217673978456407 | 0.2373337871072032 | 0.6937623481168823 |
| 0.0844814556650385 | 0.8282963418322064 | 0.9146456401097620 |
| 0.9155185593349587 | 0.1717036731677983 | 0.0853543748902430 |
| 0.4155185593349592 | 0.6717036581677936 | 0.9146456401097620 |
| 0.5844814406650413 | 0.3282963418322064 | 0.0853543748902430 |
| 0.1717036731677983 | 0.5844814406650413 | 0.4146456401097617 |
| 0.8282963418322064 | 0.4155185593349592 | 0.5853543598902380 |
| 0.3282963418322064 | 0.9155185593349587 | 0.4146456401097617 |
| 0.6717036581677936 | 0.0844814556650385 | 0.5853543598902380 |
| 0.1291960792257675 | 0.8708355580172188 | 0.8572566569515423 |
| 0.8708039137742356 | 0.1291644269827831 | 0.1427433130484549 |
| 0.3708039137742351 | 0.6291644419827812 | 0.8572566569515423 |
| 0.6291960862257644 | 0.3708355580172193 | 0.1427433130484549 |
| 0.1291644269827831 | 0.6291960862257644 | 0.3572566869515452 |
| 0.8708355580172188 | 0.3708039137742351 | 0.6427433430484577 |
| 0.3708355580172193 | 0.8708039137742356 | 0.3572566869515452 |
| 0.6291644419827812 | 0.1291960792257675 | 0.6427433430484577 |
| 0.4336736858893239 | 0.1719464351433451 | 0.7968199455110498 |
| 0.5663263141106837 | 0.8280535648566546 | 0.2031800694889475 |
| 0.0663263141106762 | 0.3280535648566549 | 0.7968199455110498 |
| 0.9336736858893163 | 0.6719464351433454 | 0.2031800694889475 |
| 0.8280535648566546 | 0.9336736858893163 | 0.2968199455110502 |
| 0.1719464351433451 | 0.0663263141106762 | 0.7031800544889502 |
| 0.6719464351433454 | 0.5663263141106837 | 0.2968199455110502 |
| 0.3280535648566549 | 0.4336736858893239 | 0.7031800544889502 |
| 0.4476717949558430 | 0.1919132668946719 | 0.9152858537264861 |
| 0.5523282050441563 | 0.8080867031053256 | 0.0847141312735233 |
| 0.0523282050441568 | 0.3080867331053281 | 0.9152858537264861 |
| 0.9476717949558437 | 0.6919132968946744 | 0.0847141312735233 |
| 0.8080867031053256 | 0.9476717949558437 | 0.4152858537264789 |
| 0.1919132668946719 | 0.0523282050441568 | 0.5847141462735139 |
| 0.6919132968946744 | 0.5523282050441563 | 0.4152858537264789 |
| 0.3080867331053281 | 0.4476717949558430 | 0.5847141462735139 |
| 0.1431637426924825 | 0.9030691564810096 | 0.2059563712733100 |
| 0.8568362723075226 | 0.0969308285189926 | 0.7940436137266924 |
| 0.3568362723075226 | 0.5969308435189904 | 0.2059563712733100 |
| 0.6431637276924774 | 0.4030691564810099 | 0.7940436137266924 |
| 0.0969308285189926 | 0.6431637276924774 | 0.7059563862733076 |
| 0.9030691564810096 | 0.3568362723075226 | 0.2940436137266924 |
| 0.4030691564810099 | 0.8568362723075226 | 0.7059563862733076 |
| 0.5969308435189904 | 0.1431637426924825 | 0.2940436137266924 |
| 0.4000493227432765 | 0.1415595404213349 | 0.8609836675502277 |
| 0.5999506772567232 | 0.8584404295786628 | 0.1390163474497702 |
| 0.0999506772567234 | 0.3584404595786580 | 0.8609836675502277 |
| 0.9000493227432768 | 0.6415595704213372 | 0.1390163474497702 |
| 0.8584404295786628 | 0.9000493227432768 | 0.3609836675502274 |
| 0.1415595404213349 | 0.0999506772567234 | 0.6390163324497723 |
| 0.6415595704213372 | 0.5999506772567232 | 0.3609836675502274 |
| 0.3584404595786580 | 0.4000493227432765 | 0.6390163324497723 |
| 0.3232250752562813 | 0.0700357210901750 | 0.0365374248944633 |
| 0.6767748947437231 | 0.9299642519098190 | 0.9634625711055358 |
| 0.1767749247437185 | 0.4299642819098219 | 0.0365374248944633 |
| 0.8232251052562769 | 0.5700357480901810 | 0.9634625711055358 |
| 0.9299642519098190 | 0.8232251052562769 | 0.5365374288944642 |

| | | |
|---|---|---|
| 0.0700357210901750 | 0.1767749247437185 | 0.4634625711055360 |
| 0.5700357480901810 | 0.6767748947437231 | 0.5365374288944642 |
| 0.4299642819098219 | 0.3232250752562813 | 0.4634625711055360 |
| 0.1098771767039883 | 0.8632144126304283 | 0.0676681446780291 |
| 0.8901227932960161 | 0.1367855723695670 | 0.9323318553219708 |
| 0.3901228232960116 | 0.6367855873695717 | 0.0676681446780291 |
| 0.6098772067039839 | 0.3632144126304280 | 0.9323318553219708 |
| 0.1367855723695670 | 0.6098772067039839 | 0.5676681446780292 |
| 0.8632144126304283 | 0.3901228232960116 | 0.4323318553219709 |
| 0.3632144126304279 | 0.8901227932960161 | 0.5676681446780292 |
| 0.6367855873695717 | 0.1098771767039883 | 0.4323318553219709 |
| 0.2996691250504578 | 0.0416076121617825 | 0.8723188839723161 |
| 0.7003308749495415 | 0.9583923858382242 | 0.1276811310276817 |
| 0.2003308749495420 | 0.4583923858382173 | 0.8723188839723161 |
| 0.7996691250504585 | 0.5416076141617758 | 0.1276811310276817 |
| 0.9583923858382242 | 0.7996691250504585 | 0.3723188839723158 |
| 0.0416076121617825 | 0.2003308749495420 | 0.6276811160276839 |
| 0.5416076141617758 | 0.7003308749495415 | 0.3723188839723158 |
| 0.4583923858382173 | 0.2996691250504578 | 0.6276811160276839 |
| 0.2577998875918425 | 0.0032566275091291 | 0.9668399969111509 |
| 0.7422000824081616 | 0.9967433424908611 | 0.0331599770888541 |
| 0.2422001124081573 | 0.4967433724908710 | 0.9668399969111509 |
| 0.7577999175918384 | 0.5032566575091389 | 0.0331599770888541 |
| 0.9967433424908682 | 0.7577999175918384 | 0.4668400269111463 |
| 0.0032566275091291 | 0.2422001124081573 | 0.5331600030888491 |
| 0.5032566575091389 | 0.7422000824081616 | 0.4668400269111463 |
| 0.4967433724908710 | 0.2577998875918425 | 0.5331600030888491 |
| 0.1500205950213397 | 0.8977267006950447 | 0.9824670298651057 |
| 0.8499793749786574 | 0.1022732993049549 | 0.0175329501349000 |
| 0.3499794049786600 | 0.6022732993049553 | 0.9824670298651057 |
| 0.6500206250213426 | 0.3977267006950451 | 0.0175329501349000 |
| 0.1022732993049549 | 0.6500206250213426 | 0.4824670598651007 |
| 0.8977267006950447 | 0.3499794049786600 | 0.5175329701348943 |
| 0.3977267006950451 | 0.8499793749786574 | 0.4824670598651007 |
| 0.6022732993049553 | 0.1500205950213397 | 0.5175329701348943 |
| 0.1752723005771718 | 0.9307301574877053 | 0.1364171894152803 |
| 0.8247276844228306 | 0.0692698575122923 | 0.8635827955847147 |
| 0.3247276844228306 | 0.5692698425122947 | 0.1364171894152803 |
| 0.6752723155771694 | 0.4307301574877055 | 0.8635827955847147 |
| 0.0692698575122923 | 0.6752723155771694 | 0.6364172044152853 |
| 0.9307301574877053 | 0.3247276844228306 | 0.3635827955847149 |
| 0.4307301574877055 | 0.8247276844228306 | 0.6364172044152853 |
| 0.5692698425122947 | 0.1752723005771718 | 0.3635827955847149 |
| 0.2823508750876392 | 0.0333818388300495 | 0.1187544792340569 |
| 0.7176491249123538 | 0.9666181551699498 | 0.8812455057659455 |
| 0.2176491249123610 | 0.4666181551699499 | 0.1187544792340569 |
| 0.7823508750876462 | 0.5333818448300502 | 0.8812455057659455 |
| 0.9666181551699498 | 0.7823508750876462 | 0.6187544942340545 |
| 0.0333818388300495 | 0.2176491249123610 | 0.3812455057659452 |
| 0.5333818448300502 | 0.7176491249123538 | 0.6187544942340545 |
| 0.4666181551699499 | 0.2823508750876392 | 0.3812455057659452 |

Total energy = −825.52517965 eV
